# Cystotomy with or without fibrinogen clot removal for refractory cystoid macular edema secondary to branch retinal vein occlusion

**DOI:** 10.1038/s41598-021-88149-z

**Published:** 2021-04-19

**Authors:** Hiroko Yamada, Hisanori Imai, Akira Tetsumoto, Mayuka Hayashida, Keiko Otsuka, Akiko Miki, Sentaro Kusuhara, Makoto Nakamura

**Affiliations:** 1 Division of Ophthalmology, Department of Surgery-Related, Kobe University Graduate School of Medicine, 7-5-2 Kusunoki-cho, Chuo-ku, Kobe, 650-0017 Japan; 2 Kobe Kaisei Hospital, 3-11-15 Shinohara Kitamachi, Nada-ku, Kobe, 657-0068 Japan

**Keywords:** Retinal diseases, Outcomes research

## Abstract

To demonstrate the long-term effect of cystotomy with or without fibrinogen clot removal for treatment-resistant cystoid macular edema (CME) secondary to branch retinal vein occlusion (BRVO). Retrospective clinical study. We retrospectively analyzed medical records of 22 eyes of 22 patients with treatment-resistant CME secondary to BRVO with 12 months observation after cystotomy with or without fibrinogen clot removal. Patients included 11 women and 11 men. The mean ± SD age was 72.7 ± 10.2 years. LogMAR-converted best corrected visual acuity (BCVA) was statistically better at 12 months after surgery (0.30 ± 0.30) than preoperative BCVA (0.39 ± 0.27) (p = 0.01). The central sensitivity of microperimetry (dB) was maintained during follow-up (preoperative sensitivity: 25.4 ± 4.1, postoperative sensitivity at 12 months after the surgery: 25.9 ± 4.2, p = 0.69). Twelve months after surgery, there was a significant improvement in the central retinal thickness (CRT) on optical coherence tomography (OCT) (303.7 ± 80.1) (μm) compared with the preoperative CRT (524.2 ± 114.8) (p < 0.01). In 12 months, CME recurred in 3 of 22 eyes. The preoperative reflectivity in cystoid cavity on OCT was significantly higher in patients with fibrinogen clot removal (n = 5) than in patients without fibrinogen clot removal (n = 17) (p < 0.01). For treatment-resistant CME secondary to BRVO, Cystotomy with or without fibrinogen clot removal may be one of the treatment options.

## Introduction

Second only to diabetic retinopathy (DR), retinal vein occlusion (RVO) is one of the most common retinal vascular disorders that threaten vision^1,2^. Approximately 16 million people are reported to have RVO according to population surveys of the United States, Europe, Asia, and Australia^3^. RVO results in a decline of visual acuity due to complications such as vitreous hemorrhage, epiretinal membrane formation, tractional retinal detachment, and macular edema.

Macular edema is a pathology that occurs secondary to various vitreoretinal disorders such as RVO and DR^4^. Conventional therapies such as intravitreal anti-vascular endothelial growth factor (VEGF) injection, intravitreal or sub-tenon triamcinolone acetonide injection, retinal photocoagulation, and pars plana vitrectomy (PPV) or combinations of such therapies have increased the proportion of successful anatomical and functional outcomes^5^. However, treatment-resistant refractory foveal cystoid macular edema (rfCME) still exists. Thus, standard therapy alone cannot achieve complete remission of macular edema in all cases, and the pathology involved in resistance to therapy remains unknown. Therefore, a novel decisive therapy is yet to be developed for this unmet need.

Cystotomy for cystoid macular edema (CME) was first reported by Tachi et al. in 1999 as a complementary therapy to primary vitrectomy for diabetic macular edema (DME)^6^. They reported that 7 of 22 eyes had improved vision, 13 had no change, and 2 had worsened after surgery. We recently reported that the accumulation of fibrinogen in cystoid cavities of rfCME secondary to branch retinal vein occlusion (BRVO), DR, and idiopathic macular telangiectasia type 1, and the removal of fibrinogen clots with cystotomy showed postoperative functional and anatomical improvements^7,8^. These outcomes suggest the effectiveness of cystotomy and fibrinogen clot removal for rfCME secondary to BRVO, but the details have not been investigated.

This study thus aimed perform a functional and anatomical assessment of cystotomy for rfCME secondary to treatment-resistant BRVO.

## Results

**Figure 1:**
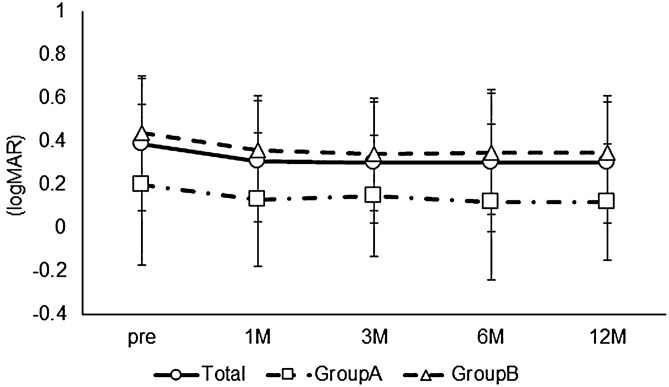
The time course change in best corrected visual acuity (BCVA). The mean preoperative BCVA (logMAR) was 0.39 ± 0.27 in all eyes, 0.20 ± 0.13 in Group A, and 0.44 ± 0.27 in Group B. At each postoperative time point, the BCVA was 0.31 ± 0.27, 0.30 ± 0.27, 0.30 ± 0.29, and 0.30 ± 0.30, respectively, in all eyes, and 0.14 ± 0.12, 0.15 ± 0.13, 0.12 ± 0.12, and 0.12 ± 0.17, respectively, in Group A, and 0.36 ± 0.28, 0.34 ± 0.28, 0.35 ± 0.31, and 0.35 ± 0.31, respectively, in Group B. In all eyes, the BCVA at each postoperative time point was statistically better than the preoperative BCVA (Friedman's test, p < 0.01; post hoc analysis, p < 0.01 at all the time points). The BCVA did not change statistically during the follow-up period in each group (Friedman's test, p = 0.30, p = 0.05, respectively).

**Figure 2:**
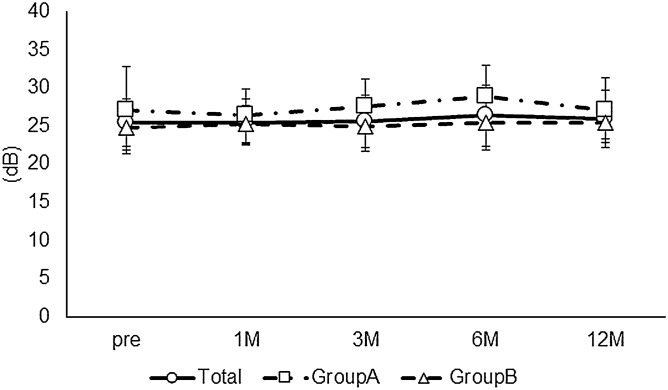
The time course change in central sensitivity on microperimetry (CS-MP3). The mean preoperative CS-MP3 (dB) was 25.4 ± 4.1 in all eyes, 27.1 ± 3.3 in Group A, and 24.8 ± 4.3 in Group B. At each postoperative time point, the CS-MP3 was 25.5 ± 3.5, 25.6 ± 4.8, 26.4 ± 5.2, and 25.9 ± 4.2, respectively, in all eyes, and 26.4 ± 3.6, 27.6 ± 2.9, 28.9 ± 2.3, and 27.1 ± 2.2, respectively, in Group A, and 25.2 ± 3.5, 24.9 ± 5.2, 25.4 ± 5.7, and 25.5 ± 4.8, respectively, in Group B. In all groups, the CS-MP3 at each postoperative time point was not statistically different from the preoperative CS-MP3 (Friedman's test, p = 0.52, p = 0.10, p = 0.90, respectively,)

**Figure 3:**
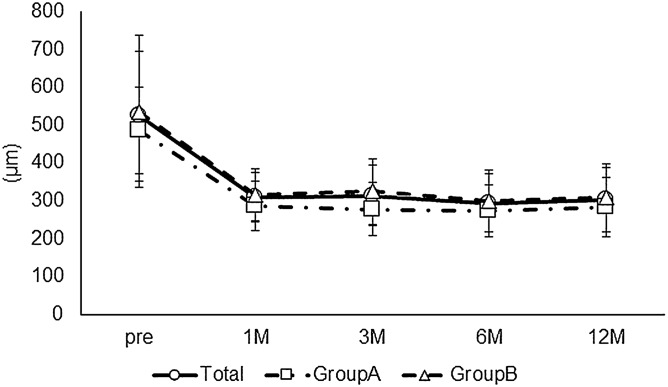
The time course change in central retinal thickness (CRT). The mean preoperative CRT (μm) was 524.2 ± 114.8 in all eyes, 486.4 ± 63.7 in Group A, and 535.4 ± 125.3 in Group B. At each postoperative time point, the CRT was 309.6 ± 65.5, 314.6 ± 66.2, 293.8 ± 52.2, and 303.7 ± 80.1, respectively, in all eyes, and 286.2 ± 57.6, 278.9 ± 23.5, 274.4 ± 17.0, and 284.2 ± 36.1, respectively, in Group A, and 316.4 ± 67.6, 325.2 ± 71.4, 299.5 ± 57.8, and 309.4 ± 89.1, respectively, in Group B. In all groups, the CRT at each postoperative time point was statistically better than the preoperative CRT ( Friedman's test, p < 0.01, p = 0.03, p < 0.01; post hoc analysis, p < 0.01, p < 0.01, p < 0.01 at all the time points, respectively).

**Figure 4:**
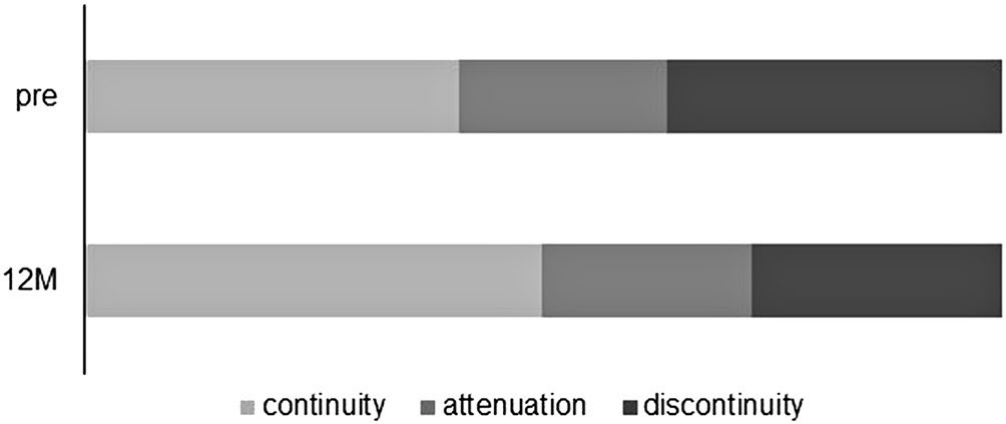
The change in perioperative external limiting membrane (ELM) status. In all eyes, the proportion of continuity, attenuation, and discontinuity was not statistically different between preoperative (9/5/8) and 12 months postoperative (11/5/6) (p = 0.90).

The long-term postoperative results of cystotomy for CME secondary to BRVO were investigated. Preoperative demographic data are described in Table 1. Eleven men and 11 women were included in this study. The mean ± SD age was 72.7 ± 10.2 years. The BCVA at each postoperative time point was significantly better than the preoperative BCVA (Friedman's test, p = 0.01; post hoc analysis, p < 0.01 at all the time points) (Fig. 1). None of the patients enrolled in this study had postoperative vision loss due to this surgical procedure. The CS-MP3 did not change statistically during the follow-up period (Friedman's test, p = 0.52) (Fig. 2). The CRT at each postoperative time point was significantly better than the preoperative CRT (Friedman's test, p < 0.01; post hoc analysis, p < 0.01 at all the time points) (Fig. 3). In terms of subfoveal ELM continuity, the disrupted or undisrupted condition of the ELM did not significantly change before and 12 months after the surgery (p = 0.90) (Fig. 4). During the follow-up period, CME relapsed in 3 of 22 eyes.

**Table 1:** Patient demographics.

	Total	Group A	Group B	p value
No. of eyes	22	5	17	-
Sex, male/female	11/11	3/2	8/9	1
Age (years), mean ± SD	72.7 ± 10.2	74.2 ± 6.2	72.3 ± 11.2	0.72
Preoperative BCVA (logMAR), mean ± SD	0.39 ± 0.27	0.20 ± 0.13	0.44 ± 0.27	0.06
Preoperative CS-MP3(dB), mean ± SD	25.4 ± 4.1	27.1 ± 3.3	24.8 ± 4.3	0.35
Preoperative CRT(μm), mean ± SD	524.2 ± 114.8	486.4 ± 63.7	535.4 ± 125.3	0.42
Reflectivity level in cystoid cavity, mean ± SD	12.93 ± 13.32	30.1 ± 6.9	7.7 ± 9.8	< 0.01
Preoperative ELM status, (cont/ atte/ discont)	9/5/8	1/3/1	8/2/7	0.30
Lens status, phakia/pseudophakia	9/13	2/3	7/10	1
Recurrence of CME at 12 months, (Yes/No)	3/22	0/5	3/17	1

*SD* standard deviation, *DR* diabetic retinopathy, *RVO* retinal vein occlusion, *CRT* central retinal thickness, *BCVA* best-corrected visual acuity, *logMAR* the logarithm of the minimum angle of resolution, *CS* central sensitivity, *MP* microperimetry, *ELM* external limiting membrane, *cont* continuous, *ate* attenuation, *discount* discontinuous, *CME* cystoid macular edema.

**Figure 5:**
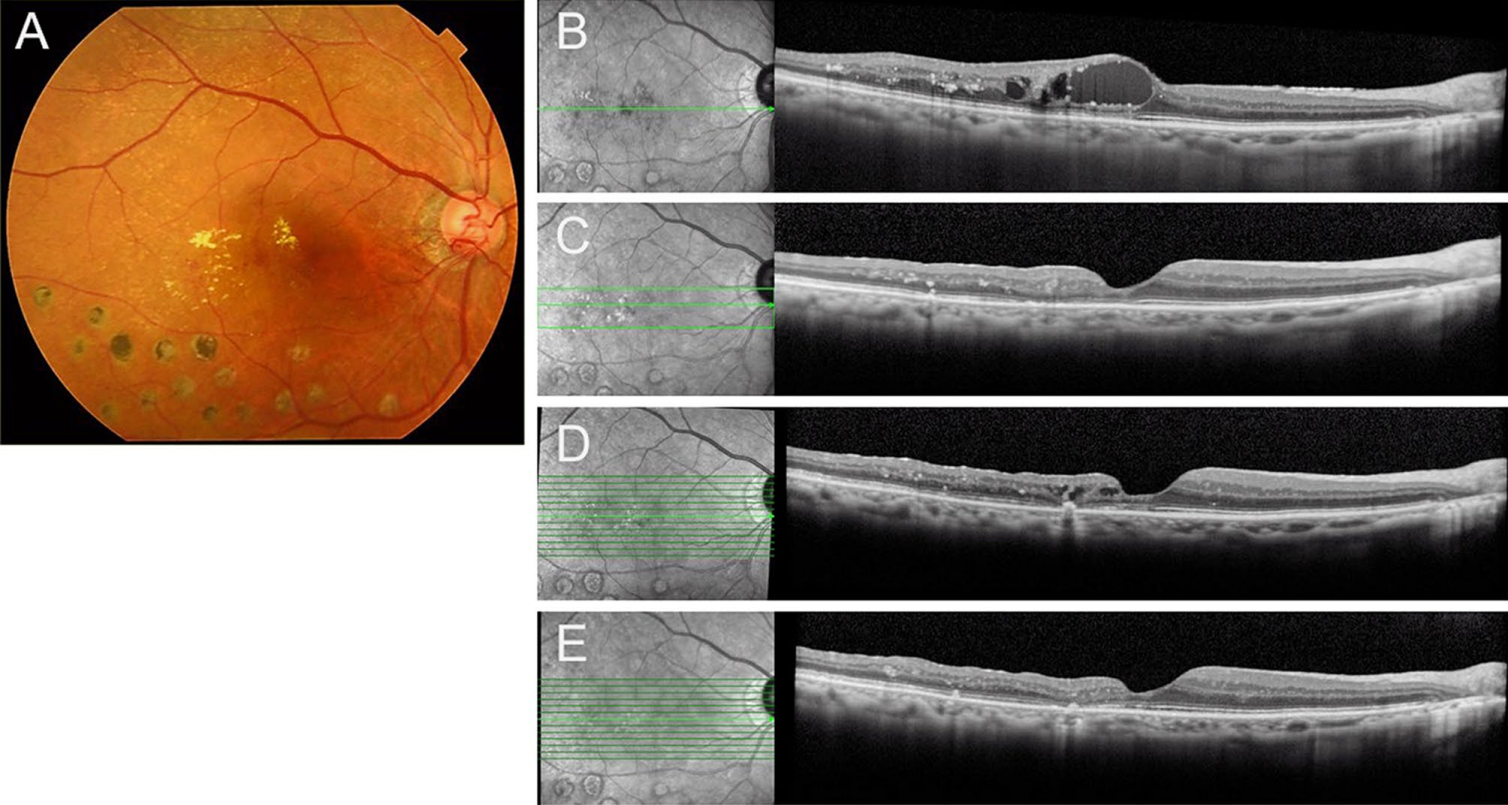
An 86-year -old woman was referred to us for the treatment of prolonged resistant cystoid macular edema (CME) secondary to branch retinal vein occlusion in the right eye. At the initial visit, best corrected decimal visual acuity (BCVA) was 0.6. A fundus examination revealed a CME and postlaser chorioretinal scars (**A**). The foveal cystoid cavity showed higher reflectivity compared to the vitreous on optical coherence tomography finding (**B**). Cystotomy and fibrinogen clot removal were performed for the treatment of CME. CME subsided immediately after surgery with no recurrence during the follow-up period of 12 months (**C** 1 month, **D** 6 months, **E** 12 months after the surgery).The patient had BCVA of 1.0 at the last medical examination.

Next, the long-term postoperative results of cystotomy were compared between patients in whom the fibrinogen clot was removed during the surgery (Group A) and patients in whom the clot was not removed (Group B). Preoperative demographic data of both the groups are described in Table 1. There were 5 eyes and 17 eyes in each group, respectively. Group A and Group B comprised of 3 and 8 men, respectively (p = 1). The mean ± SD age (years) in each group was 74.2 ± 6.2 and 72.3 ± 11.2, respectively (p = 0.72). In Group A and B, the preoperative BCVA (logMAR) was 0.20 ± 0.13 and 0.44 ± 0.27 (p = 0.06), CS-MP3 (dB) was 27.1 ± 3.3 and 24.8 ± 4.3 (p = 0.35), and CRT (µm) was 486.4 ± 63.7 and 535.4 ± 125.3 (p = 0.42), respectively. The preoperative reflectivity level in cystoid space in Group A (30.1 ± 6.9) was significantly higher compared with that in Group B (7.7 ± 9.8) (p < 0.01). There was no statistical difference in preoperative continuity of the ELM between groups (p = 0.30). CME did not relapse in Group A. However, CME relapsed in 3 eyes in Group B (p = 1). The BCVA did not change statistically during the follow-up period in each group (Friedman's test, p = 0.30, p = 0.05, respectively) (Fig. 1). The CS-MP3 did not change statistically during the follow-up period in each group (Friedman's test, p = 0.10, p = 0.90, respectively) (Fig. 2). The CRT at each postoperative time point was significantly thinner than the preoperative CRT in each group (Friedman's test, p = 0.03, p < 0.01; post hoc analysis, p < 0.01, p < 0.01 at all the time points, respectively) (Fig. 3). Figure 5 and the supplemental digital content shows a representative case in which cystotomy and fibrinogen clot removal were markedly effective (see Video S1, Supplemental Digital Content, which demonstrates the method for the excision of the component in the same case of Fig. 5).

The results of the preoperative blood examination were not different between the two groups (Table 2).

**Table 2:** Results of preoperative blood examination.

	Total	Group A	Group B	p value
BS (mg/dL), mean ± SD	120.9 ± 41.3	146.0 ± 67.2	114.9 ± 33.0	0.18
HbA1c (%), mean ± SD	6.23 ± 0.46	6.27 ± 0.80	6.22 ± 0.41	0.87
SBP (mmHg) , mean ± SD	128.3 ± 15.7	121.6 ± 17.6	130.3 ± 15.1	0.29
DBP (mmHg) , mean ± SD	67.7 ± 9.2	68.4 ± 2.6	67.5 ± 10.5	0.85
PT-INR, mean ± SD	1.06 ± 0.17	0.99 ± 0.07	1.08 ± 0.19	0.29
APTT (s), mean ± SD	29.97 ± 3.16	27.84 ± 1.09	30.59 ± 3.31	0.09

*BS* blood sugar, *HbA1c* hemoglobin A1c, *SBP* systolic blood pressure, *DBP* diastolic blood pressure, *PT-INR* prothrombin time-international normalized ratio, *APTT* activated partial thromboplastin time.

## Discussion

The efficacy of cystotomy for rfCME has only been demonstrated for rfCME secondary to DM^6,9^. Our study demonstrated that cystotomy was effective for rfCME secondary to BRVO as well. Retinal microvascular damage induced by metabolic disorder associated with chronic hyperglycemia causes breakdown of the blood-retinal barrier, plasma extravasation to the intercellular space, and leaking edema, as described in DR^10^. On the contrary, after venous occlusion in BRVO, capillaries and post-capillary venules in the drained area are gradually dilated, and due to the breakdown of the blood-retinal barrier induced in this process, plasma extravasation to the intercellular space and leaking edema occur, as shown in animal experiments^11^. Thus, although the mechanism of retinal edema is different in both diseases, in both diseases, lack of a fluid outflow mechanism via the capillary barrier in the fovea and weak intercellular bonding between Müller cells result in the formation of large cystoid edema along the Henle fiber (outer plexiform) layer^12^. As such, the mechanisms of onset of foveal CME are common in these two diseases. We found a high efficacy of cystotomy in rfCME following BRVO in the present study, which is in agreement with previously reported findings for rfCME following DM^6,9^. This result may suggest that cystotomy is not only effective for rfCME following DR, but also for CME caused by any microvascular retinal disorder, including BRVO. However, further investigations for other diseases are warranted.

Among the patients who were indicated for surgery in the present study, fibrinogen clots could be removed in 5 of 22 eyes. It is interesting to note that fibrinogen clots form in the cystoid space in CME following RVO as well. In patients with DR, blood and intravitreal fibrinogen concentration are significantly higher than in healthy patients^13–17^. Ocular autopsies have shown intraretinal fibrinogen exudation in DR^17^, suggesting links with the onset and progression of the disease. However, blood fibrinogen concentration in patients with RVO has also been reported to be significantly higher than that in healthy individuals^2,18–23^. It has also been reported that blood fibrinogen concentration is higher in patients with avascular regions than otherwise^24,25^. Taken together, we speculate that in vivo overproduction of fibrinogen may be associated with the onset or progression of various microvascular retinal disorders including RVO, similar to DM. This is a significant finding, in that, fibrinogen clots formed in CME are similar to those which develop in two distinct diseases of different etiologies—i.e., DM and RVO—and future investigations are needed to study the role of the presence of fibrinogen in the onset or progression of CME.

The limitations of this study are as follows. First, it is a retrospective, non-controlled pilot study conducted with a small sample size, and warrants a prospective controlled trial with a larger cohort for further investigation. In particular, the number of cases in which the fibrinogen clot was removed was very small, and there was a large difference between the number of total cases in the removed and non-removed groups. Therefore, it is difficult to conclusively evaluate the difference in therapeutic effect between the two groups, and the comparison between the groups made in this study should be considered only as reference data. Nevertheless, this study reported that cystostomy could resolve CME resistant to other therapies. Thus, our findings support the efficacy of cystotomy for rfCME secondary to BRVO. Anti-VEGF is the first-line therapy for CME secondary to BRVO; however, cystotomy may be an effective option for rfCME resistant to conventional treatments. In the future, it will be important to conduct a controlled trial of patients who undergo vitrectomy for CME resistant to various conservative therapies and subsequently undergo cystotomy and compare the outcomes with those who do not undergo further surgery.

In conclusion, cystotomy with or without fibrinogen clot removal was found to be effective for the long-term anatomical and functional improvement of refractory CME secondary to BRVO.

## Subjects and methods

Our study was performed after obtaining the approval of the Ethics Committee of Kobe University Graduate School of Medicine (approval number: 170083). The procedure conformed to the tenets of the Declaration of Helsinki. Patient inclusion criteria included surgeries performed at Kobe University Hospital from September 2014 through January 2019. In this study, informed consent was not obtained from each patient because of the retrospective, observational nature of the study. Although the need for informed consent was waived by the Ethics Committee of Kobe University Graduate School of Medicine, patients were able to withdraw consent any-time for providing information about this study, which could be accessed on the hospital homepage as an opt-out choice.

A retrospective analysis of the medical records of 22 eyes of 22 patients who underwent PPV for the purpose of cystotomy and/or fibrinogen clot removal was performed. This study was carried out with minor modifications based on the protocol of our previous study^9^. Surgery was introduced when CME secondary to BRVO was resistant to all existing conventional treatments, such as an intravitreal anti-VEGF injection, a sub-tenon triamcinolone acetonide injection (STA), direct photocoagulations for microaneurysms, and PPV with internal limiting membrane peeling. Intravitreal anti-VEGF administration was given at least 3 times to all patients prior to this study. The average number of doses of anti-VEGF drug is 5.9 ± 5.1. STA was performed at the discretion of the attending physician and at the patient's request. It was performed on 9 out of 22 eyes. When it was judged that the causative microaneurysm could be identified from the observation of the fundus and the findings of optical coherence tomography, direct photocoagulation of the microaneurysm was performed. It was done in 12 out of 22 eyes. Due to poor response to conventional therapies such as anti-VEGF therapy, STA, and retinal photocoagulation described above, all 22 patients underwent PPV with internal limiting membrane peeling prior to this study. The Patients who had been followed-up at least for 12 months after surgery were enrolled.

The following clinical variables were analyzed: sex, age, best corrected decimal visual acuity (BCVA), central sensitivity (CS) on microperimetry (MP-3; NIDEK CO., LTD.) (CS-MP3) (dB), lens status, presence or absence of fibrinogen clot removal during surgery, presence or absence of CME recurrence, and the parameters determined by optical coherence tomography (OCT) including central retinal thickness (CRT) (µm), reflectivity of cystoid cavity of CME, and continuity of the external limiting membrane (ELM).

The BCVA, CS-MP3, and CRT were measured preoperatively, and 1 month, 3 months, 6 months, and 12 months after the surgery.

Microperimetry was performed on all patients with MP-3 in a dark room with pupillary dilation. We used the macula 12S2 program for this study. A white stimulus with Goldmann III in size and duration of 200 ms was projected onto a white background at 31.4 asb. The stimulus dynamic range for the MP-3 was 34 dB, and the light threshold was determined by a 4–2 staircase strategy. The mean retinal sensitivity of the central 9 points, which corresponds to a diameter of 4°, was defined as CS-MP3 and used for the statistical analysis.

The CRT, reflectivity of cystoid cavity of CME, and continuity of subfoveal ELM were assessed using spectral domain OCT (Spectralis HRA + OCT; Heidelberg Engineering, Heidelberg, Germany). For the evaluation of cystoid cavity reflectivity, six-line radial scans (30 degrees) centered on the presumed fovea were obtained and the inverted grayscale vertical images were used for further investigation. The cystoid cavities in OCT images were circumscribed manually followed by measurement of the average reflectivity in this area using the Image J software (version 1.48, National Institutes of Health, USA). We used the reflectivity levels of the vitreous cavity as the standard in each image. The reflectivity value of the cystoid cavity relative to the vitreous cavity was calculated as an arbitrary unit (AU).

The results of preoperative blood examination were also assessed. The inspection items were as follows: blood glucose (BS) (mg/dL), HbA1c (%), systolic blood pressure (mmHg), diastolic blood pressure (mmHg), prothrombin time-international normalized ratio (PT-INR), activated partial thromboplastin time (APTT) (s).

### Statistical methods

The statistical analysis was conducted based on the same method of our previous study^9^. The unpaired t-test for continuous variables, and the chi-square test and Fisher’s exact probability test for dichotomous variables were used to compare the parameters listed above. The Friedman's test, followed by post hoc analysis using Wilcoxon t-test with Bonferroni correction, was used to evaluate the time course changes in BCVA, CS-MP3, and CRT in each group.

The Landolt-decimal BCVA was converted to the logarithm of the minimal angle of resolution (logMAR) for statistical analysis. Statistical analyses were performed using statistical software (SPSS, version 24.0; IBM Corporation, Armonk, NY). Statistical significance was inferred for p < 0.05.

### Surgical procedures

All surgeries were performed as previously reported^7^. In summary, standard 27-gauge PPV with a non-contact wide-angle viewing system (Resight; Carl Zeiss Meditec AG, Jena, Germany) was performed by one experienced surgeon (HI) in the same operating room. The Constellation Vision System (Alcon Laboratories, Inc., Fort Worth, TX, USA) was used for the surgery. Before vitrectomy, phacoemulsification and intraocular lens implantation with 2.4 mm bent transconjunctival single-plane sclera-corneal or clear corneal incision were performed using the same machine for all phakic eyes. First, by using 27-gauge microforceps or microscissors, the outer wall of subfoveal cystoid space was torn or incised. Next, if the surgeon could visually confirm the presence of an exposed fibrinogen clot in the cyst space, it was directly grasped and excised using 27-gauge microforceps. STA and/or intravitreal triamcinolone acetonide injection immediately at the end of the surgery were not performed on any case in this study.

In actual surgery, after the de-roofing of the cystoid cavity, the surgeon could check the presence of fibrinogen clot visually. Fibrinogen clot is not completely transparent and has a different color and reflection from other retinal tissues^9^, making it easy to identify. If the surgeon was unable to identify the presence of a fibrinogen clot visually, no manipulation was done in the cystoid cavity.

## Supplementary Information

Supplementary Video 1.

## Data Availability

The datasets generated during and/or analyzed during the current study are available from H. Imai, the corresponding author on reasonable request.
